# The *AaCBF4-AaBAM3.1* module enhances freezing tolerance of kiwifruit (*Actinidia arguta*)

**DOI:** 10.1038/s41438-021-00530-1

**Published:** 2021-05-01

**Authors:** Shihang Sun, Chungen Hu, Xiujuan Qi, Jinyong Chen, Yunpeng Zhong, Abid Muhammad, Miaomiao Lin, Jinbao Fang

**Affiliations:** 1 Key Laboratory for Fruit Tree Growth, Development and Quality Control, Zhengzhou Fruit Research Institute, Chinese Academy of Agricultural Sciences, Zhengzhou, 450009 China; 2 Key Laboratory of Horticultural Plant Biology (Ministry of Education), College of Horticulture and Forestry Science, Huazhong Agricultural University, Wuhan, 430070 China

**Keywords:** Abiotic, Signal processing

## Abstract

Beta-amylase (BAM) plays an important role in plant resistance to cold stress. However, the specific role of the *BAM* gene in freezing tolerance is poorly understood. In this study, we demonstrated that a cold-responsive gene module was involved in the freezing tolerance of kiwifruit. In this module, the expression of *AaBAM3.1*, which encodes a functional protein, was induced by cold stress. *AaBAM3.1*-overexpressing kiwifruit lines showed increased freezing tolerance, and the heterologous overexpression of *AaBAM3.1* in *Arabidopsis thaliana* resulted in a similar phenotype. The results of promoter GUS activity and *cis*-element analyses predicted *AaCBF4* to be an upstream transcription factor that could regulate *AaBAM3.1* expression. Further investigation of protein-DNA interactions by using yeast one-hybrid, GUS coexpression, and dual luciferase reporter assays confirmed that AaCBF4 directly regulated *AaBAM3.1* expression. In addition, the expression of both *AaBAM3.1* and *AaCBF4* in kiwifruit responded positively to cold stress. Hence, we conclude that the *AaCBF-AaBAM* module is involved in the positive regulation of the freezing tolerance of kiwifruit.

## Introduction

Cold stress is a kind of abiotic stress that can considerably limit both plant growth and yield and can determine the geographic distribution of plants^1^. Therefore, freezing tolerance acts as an important quantitative trait and has received considerable amounts of attention for the past several decades^2–5^. In model plant species, researchers have identified complex signaling networks consisting of cold sensors, secondary messengers, transcription factors (TFs), phytohormones and functional proteins^6–9^. Low temperature is also a common factor that significantly hinders the quality and productivity of kiwifruit. In the last twenty years, kiwifruit has often suffered from cold damage worldwide, which has had a large influence on the kiwifruit industry. However, there have been few studies on the freezing tolerance of kiwifruit. Hence, there is an urgent need to understand the freezing tolerance mechanisms of kiwifruit under cold stress to develop novel genetic resources affording strong freezing tolerance^10^.

Cold stress can cause two major types of injury: osmotic stress and oxidative stress^11,12^. When plants are subjected to cold stress, their cells ultimately experience osmotic stress due to the formation of ice crystals^13^. Under osmotic stress, the changes in the accumulation of compatible solutes are the most prominent alterations observed among the physiological and biochemical characteristics of plants^14^. Proline and soluble sugars, which act as compatible solutes that are involved in multiple metabolic pathways, are produced and accumulate under cold stress^15^. Oxidative stress causes the excessive accumulation of reactive oxygen species (ROS) due to an imbalance between their production and scavenging^16^. ROS cause the denaturation of lipids, proteins and nucleic acids, which can lead to further metabolic disorders^17^. Both osmotic and oxidative stresses develop simultaneously by causing synergistic effects under cold stress. Moreover, it is gradually becoming understood that compatible solutes have important effects on ROS scavenging and enhanced freezing tolerance^18^. To sense and adapt to cold stress, plants have developed sophisticated and efficient mechanisms to protect themselves from cold injury, and the expression of a number of genes is induced in response to low temperature to provide resistance to both osmotic stress and oxidative stress^19^.

In the early 1980s, we had a poor understanding of beta-amylases (BAMs), which are considered to be dispensable storage proteins^20,21^. We have now acquired detailed information about BAMs, which have been shown to play key roles in response to abiotic stress by degrading starch^22^. BAMs are members of glycosyl hydrolase family 14 (PF01373) and have two typical catalytic sites at the N-terminus and in a central location; thus, these enzymes can hydrolyze 1,4-alpha-glucosidic linkages within starch^23^. BAMs are considered to be the mediators that degrade starch into downstream soluble sugars and play a pivotal role in the accumulation of soluble sugars under cold stress. Moreover, soluble sugars derived from the degradation of starch are transferred from chloroplasts to the cytoplasm and can then participate in the energy metabolism pathway to resist cold damage^24,25^. In perennial plants, previous studies have indicated that the expression level of *BAM* mRNA and the activity of BAMs could be elevated in pear, blueberry, orange and tea trees under cold stress^26–29^. BAM activity has also been shown to increase in potato under low temperature^30^. The idea that *BAM* is involved in the cold stress response was further verified in poplar, where *CBF1* is the upstream regulatory gene of *BAM*^31^. In *Arabidopsis thaliana* (*A. thaliana*), nine BAM family members have been identified in the genome: BAM1 to BAM9^32^. *A. thaliana* BAMs can be divided into two categories based on whether they show catalytic activity: BAM1-BAM3, BAM5 and BAM6 are catalytically active, whereas BAM4 and BAM7-BAM9 are proposed to be catalytically inactive. Many studies have demonstrated that *BAM3* is transcriptionally induced in response to low temperature, which indicates that cold stress might accelerate starch degradation^33^. However, the suppression of *StBAM1* expression results in only low BAM activity and freezing tolerance, indicating that *StBAM1* plays a small role in starch degradation, although it exhibits BAM catalytic activity^34^. In addition, the *AtBAM3* gene orthologs of various plant species are responsible for soluble sugar accumulation and enhanced freezing tolerance. These findings show that within the plant genome, *AtBAM3* and its orthologous genes exhibit the same function in the enhancement of freezing tolerance by degrading starch. Although the *BAM*s of *A. thaliana* have been characterized, their functions in freezing tolerance remain mostly unknown. Moreover, the available information about the functions of *BAM*s in perennial plants is still inadequate.

Over the past few decades, increasing amounts of attention have been focused on TFs. Among TFs, those encoded by C-repeat-binding factor (*CBF*) genes are thought to be mainly involved in the response to cold stress^35,36^. Furthermore, the CBF cold response pathway has been indicated to play a key role in the enhancement of freezing tolerance. Among the cold-responsive genes of *A. thaliana*, 12% can be assigned to the CBF regulon^37^. *AtCBF1*, *AtCBF2,* and *AtCBF3* (also referred to as *AtDREB1b*, *AtDREB1c,* and *AtDREB1a*, respectively) have been functionally identified in *A. thaliana*^38^. The mRNA expression of *AtCBF1*, *AtCBF2*, and *AtCBF3* has been reported to be quickly induced under cold stress, after which the encoded proteins recognize C-repeat/dehydration-responsive (CRT/DRE) *cis*-elements in the promoters of COLD-REGULATED (COR) genes and bind to these motifs. It has been determined that *CBF* expression induces the expression of a large number of COR genes, which results in constitutively enhanced freezing tolerance^39^. In contrast, the silencing of the *AtCBF1*, *AtCBF2*, and *AtCBF3* genes decreases the mRNA expression of COR genes with CRT/DRE elements, which leads to weakened freezing tolerance of plants^40^. Taken together, the above findings suggest that *CBF* genes are involved in extensive cold regulatory networks and play an important role in the response to cold stress.

The *Actinidia* genus includes 54 species and 21 varieties, all of which have varying freezing tolerance abilities^41,42^. Both *Actinidia chinensis* and *Actinidia deliciosa* are currently extensively cultivated worldwide, but these two species show very poor tolerance to −13 °C temperatures during winter, which causes severe damage to plants^43^. *Actinidia. arguta*, a prominent species within *Actinidia*, can survive at a temperature of −40 °C^42^. The variable freezing tolerance abilities within the *Actinidia* genus imply that large genetic variations exist in the kiwifruit genome. Therefore, the identification of cold-responsive genes may allow the detailed molecular mechanisms underlying the enhanced freezing tolerance abilities of kiwifruit to be further explored for molecular breeding purposes.

In this work, we identified the *A. arguta AaBAM3.1* gene, which was significantly expressed in response to cold treatment. *AaBAM3.1***-**overexpressing lines were generated in both kiwifruit (“Hongyang”) and *A. thaliana*, and the freezing tolerance of the transgenic plants was enhanced. The promoter region of *AaBAM3.1* in transgenic *A. thaliana* and its predicted *cis*-elements also responded to cold treatment, which indicated that AaCBF4 may be a candidate for the upstream regulation of *AaBAM3.1*. Through protein-DNA interaction assays, we found that AaCBF4 directly regulated *AaBAM3.1* in kiwifruit. Hence, we concluded that kiwifruit *AaBAM3.1*, which is positively regulated by AaCBF4, is a cold**-**responsive gene that can enhance freezing tolerance.

## Results

### Cloning of *AaBAM3.1* and its sequence analysis

In a previous study, we cloned and identified a cold**-**induced *BAM* gene (GenBank Accession No. MT263012) from *A. arguta*. *AaBAM3.1* had a full**-**length ORF of 1644 bp flanking a 1363 bp promoter^23^. *AaBAM3.1* encoded a protein of 548 amino acids with a 61.74 kDa molecular weight, and the theoretical isoelectric point of AaBAM3.1 was 8.76. A maximum likelihood tree was established using 14 BAM protein sequences. The BAM sequences from different plant species clustered into two main groups, I and II. AaBAM3.1 was classified into the same group (part ‘I’, corresponding to the red region in the Fig. S4a) as AtBAM3 of *A. thaliana* (Fig. S4a). Multiple alignment revealed a highly conserved catalytic and binding site among AaBAM3.1 and 9 other BAM proteins (Fig. S4b). The conserved site contained 2 catalytic residues, Glu186 and Glu380 (black arrowheads in the Fig. S4b), and 19 substrate-binding residues (red arrowheads in the Fig. S4b).

### Time course of *AaBAM3.1* expression in *A. arguta* under cold stress

**Fig. 1:**
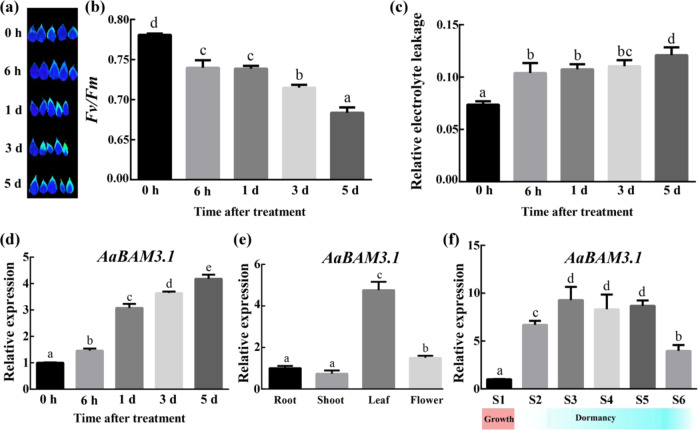
Phenotypes of *Actinidia arguta* plants under cold stress and the expression of *AaBAM3.1* in *A. arguta* in response to cold stress. **a** Chlorophyll fluorescence image. **b** Corresponding *F*_*v*_*/F*_*m*_ over the course of a 5-day low**-**temperature treatment. **c** Electrolyte leakage of *Actinidia arguta* plants after being subjected to 5 days of low**-**temperature treatment. **d** The time course of expression levels in the leaves was analyzed via RT-qPCR in response to cold stress. **e** Tissue**-**specific expression in the vegetative growth stage was analyzed via RT-qPCR. **f** *AaBAM3.1* expression in the shoots was analyzed between the growth stage and dormant stage under cold stress. S1: 15 April; S2: 15 November; S3: 7 December; S4: 23 December; S5: 15 January; S6: 15 March

Thirty**-**day**-**old *A. arguta* plants were cold subjected to 4 °C for various times. Imaging analysis demonstrated that chlorophyll fluorescence was lower in the leaves of the treated kiwifruit than in those of the untreated kiwifruit (Fig. 1a). Accordingly, the *Fv/Fm* of the leaves was progressively reduced by cold stress (Fig. 1b). The observed levels of relative electrolyte leakage (REL), a major indicator of membrane damage, indicated that membrane damage was significantly greater in the treated kiwifruit than in the untreated kiwifruit (Fig. 1c). The transcript level of *AaBAM3.1* gradually increased over 5 days of cold treatment (Fig. 1d). The results of tissue**-**specific expression analysis showed the highest expression level in the leaves during the growth stage (Fig. 1e), while the expression level in the shoots was lowest and increased in the dormant stage. When the air temperature decreased to the lowest level at the dormant stage, the expression level of *AaBAM3.1* reached the highest level (Fig. 1f). Taken together, the results indicated that *AaBAM3.1* can respond to cold stress.

### In vitro analysis of the subcellular localization of the AaBAM3.1 protein and BAM enzyme activity

**Fig. 2:**
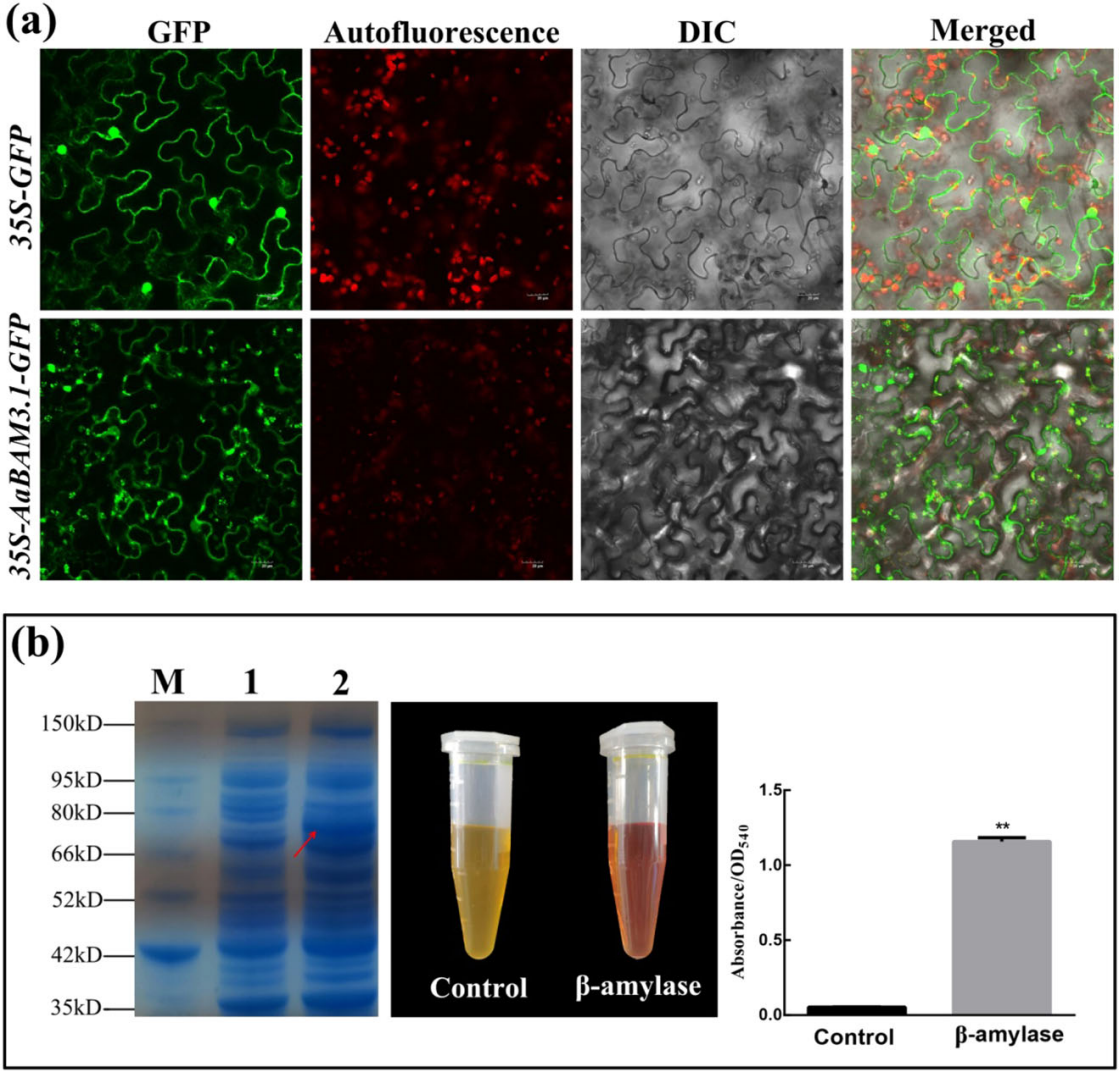
Subcellular localization of AaBAM3.1-GFP and BAM activity in pGEX4T-1. **a** Tobacco leaves were injected with the control (35S-GFP) or recombinant plasmid (35S-AaBAM3.1-GFP) and visualized under a confocal microscope. GFP imaging and autofluorescence are shown. **b** SDS-PAGE analysis of the expression of the recombinant protein in *E. coli*. The total proteins from bacteria and the purified recombinant protein from the soluble crude extract were separated via 10% SDS-PAGE and stained with Coomassie brilliant blue. M, protein size marker (15–150 kDa). Lane 1 contains proteins from uninduced cells, and lane 2 contains proteins from induced cells. The OD540 results obtained using a BAM enzyme kit showed that the recombinant protein presented enzymatic activity

The subcellular localization of AaBAM3.1 was examined via its transient expression in tobacco. We constructed an *AaBAM3.1***-**green fluorescent protein (GFP) fusion vector and used the empty vector (EV) containing GFP alone as a control. These plasmids, driven by the CaMV 35 S promoter, were injected into the tobacco leaf epidermis using the *Agrobacterium*-mediated transient expression method. GFP fluorescence was ubiquitously distributed around the whole cell in those containing the control plasmid (Fig. 2a). By contrast, the GFP signal in cells containing the recombinant plasmid was observed solely in the cytoplasm and overlapped with the red autofluorescence of chloroplasts. Overall, the results indicated that AaBAM3.1 was located in the chloroplast.

Overexpression of the AaBAM3.1**-**glutathione S**-**transferase (GST) fusion plasmid in *E*. *coli* Rosetta (DE3) cells was induced by IPTG. The total purified proteins and the induced soluble protein fraction were isolated via SDS-PAGE, and AaBAM3.1 was shown to be successfully expressed (Fig. 2b). The proteins whose expression was induced were used to analyze enzyme activity. The results indicated that the fusion protein could catalyze the transformation of starch into maltose. As expected, the control (ddH_2_O) exhibited no obvious BAM activity.

### Heterologous expression of *AaBAM3.1* enhances cold tolerance of *A. thaliana*

**Fig. 3:**
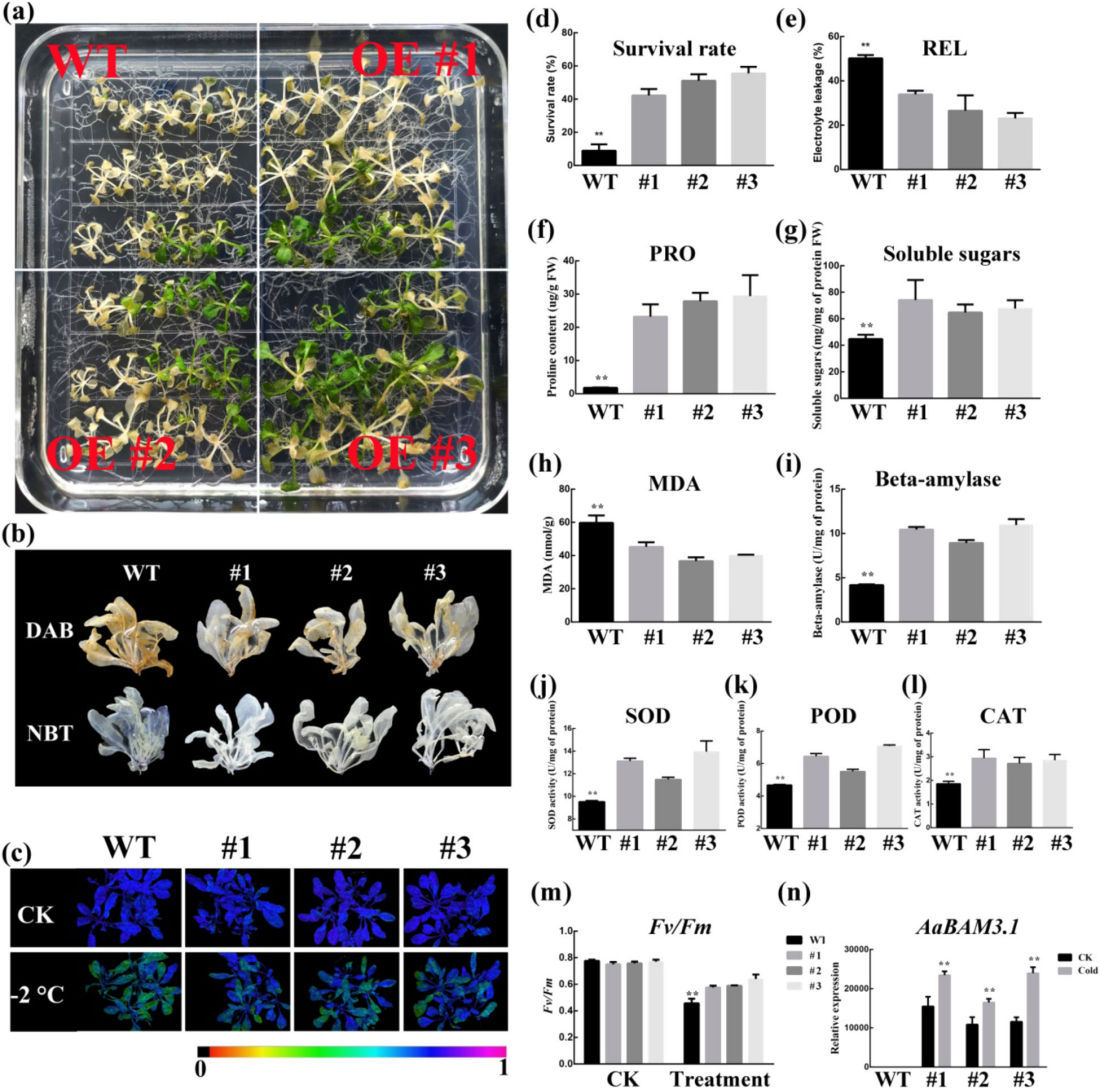
Characterization of freezing tolerance of *Arabidopsis* harboring the 35 S::AaBAM3.1 construct. **a** Phenotypes of the overexpression lines (#1, #2, and #3) and WT plants under cold stress (−2 °C for 2 h). **b** Diaminobenzidine (DAB) and nitro blue tetrazolium (NBT) staining of the overexpression lines (#1, #2, and #3) and WT plants under cold stress (−2 °C for 2 h). **c**, **m** *Fv/Fm* of *Arabidopsis* over the course of 2 h of low**-**temperature treatment. Color barcodes are shown below the images. **d**–**l** Survival rates and relative electrolyte leakage (REL), proline (PRO), soluble sugars (SS), malondialdehyde (MDA), BAM activity, superoxide dismutase (SOD) activity, peroxidase (POD) activity and catalase (CAT) activity measured under cold stress. **n** Expression of *AaBAM3.1* in *Arabidopsis* under cold stress

The transgenic and wild**-**type plants did not show obvious phenotypic differences under normal growth conditions. When 3-week-old plants were subjected to −2 °C for 2 h, the WT plants showed more severe cold injury than did the transgenic plants (Fig. 3a). After freezing stress, the average survival rate of the *AaBAM3.1***-**overexpressing plants was higher (>40%) than that of the WT plants (<20%) (Fig. 3d). Malondialdehyde (MDA) and REL levels were measured to assess membrane damage in the treated plants. We observed significantly lower levels of MDA and REL in the transgenic plants than in the WT plants under cold stress conditions (Fig. 3e, h). Previous studies have shown that the activities of antioxidant enzymes such as superoxide dismutase (SOD), peroxidase (POD), and catalase (CAT) play an important role in enhancing freezing tolerance. We also observed that the transgenic plants exhibited significantly higher SOD, POD, and CAT activities than the WT plants did under the **−**2 °C treatment (Fig. 3j, k, l). Proline (PRO) and soluble sugars (SS) are considered important compatible solutes that help plants cope with abiotic stress. After **−**2 °C treatment, the PRO and SS contents in the transgenic plants were significantly higher than those in the WT plants (Fig. 3f, g). Under the **−**2 °C treatment, in situ histochemical staining via diaminobenzidine (DAB) and nitro blue tetrazolium (NBT) revealed that H_2_O_2_ and O_2_^-^ accumulated more in the WT plants than in the transgenic plants (Fig. 3b). Chlorophyll fluorescence imaging demonstrated that the leaves of the transgenic plants maintained a higher level of chlorophyll fluorescence than did those of the WT plants under cold stress. The *Fv/Fm* values of both the transgenic and WT plants were reduced under cold stress; however, the transgenic plants displayed higher *Fv/Fm* values than did the WT plants (Fig. 3c, m). After cold stress, the relative expression of *AaBAM3.1* was higher in the treated transgenic kiwifruit plants than in the untreated transgenic plants (Fig. 3n). Taken together, the above results indicated that *AaBAM3.1* contributes to freezing tolerance by decreasing the ROS, REL and MDA levels and increasing the antioxidant enzyme activities and contents of compatible solutes.

### *AaBAM3.1* overexpression in *A. chinensis* enhances cold tolerance

**Fig. 4:**
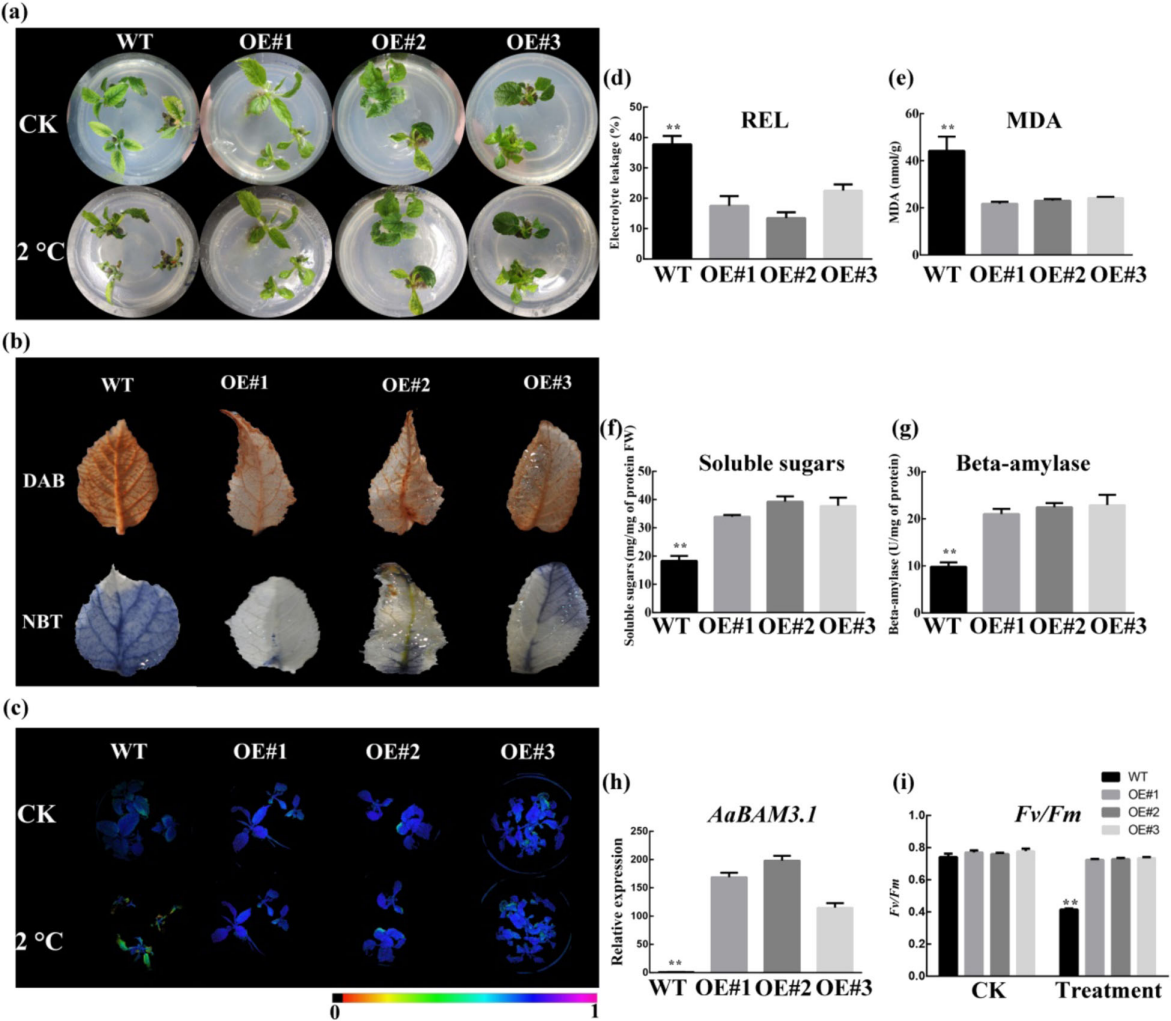
Cold tolerance characterization of *A. chinensis* harboring the 35 S::AaBAM3.1 construct. **a** Phenotypes of the overexpression lines (#1, #2, and #3) and WT plants under cold stress (2 °C for 2 h). **b** Diaminobenzidine (DAB) and nitro blue tetrazolium (NBT) staining of the overexpression lines (#1, #2, and #3) and WT plants under cold stress (2 °C for 2 h). **c**, **i** *Fv/Fm* of kiwifruit over the course of a 2 h low-temperature treatment. A colored barcode is shown below the images. **d**–**g** Relative electrolyte leakage (REL), malondialdehyde (MDA), soluble sugars (SS) and BAM activity levels were measured under cold stress. (**h**) Expression of *AaBAM3* in kiwifruit under cold stress

To confirm the roles of *AaBAM3.1* in kiwifruit, we obtained *AaBAM3.1-*overexpressing plants of *A. chinensis* cv. Hongyang by transforming explants produced from leaf strips. Three lines with high expression of *AaBAM3.1* were chosen for further studies. The cold tolerance of transgenic kiwifruit was assessed by analyzing the REL levels and *Fv/Fm*, taking WT plants as controls. After subjecting 30-day-old plants to 2 °C treatment for 1 h, the WT plants showed more severe cold injury than did the transgenic plants (Fig. 4a). Under low**-**temperature stress, the REL and MDA levels in the transgenic plants were significantly lower than those in the WT plants (Fig. 4d, e). In addition, the *Fv/Fm* was lower in the transgenic lines than in the WT (Fig. 4c, i). DAB and NBT staining indicated that the transgenic plants accumulated less H_2_O_2_ and O_2_^−^ than did the WT plants. Moreover, the levels of ROS were significantly lower in the transgenic plants than in the WT plants under cold stress (Fig. 4b). Beta-amylase activity was significantly higher in the *A. chinensis* transgenic lines than in the the WT plants after cold stress (Fig. 4g). In addition, SS levels were higher in the transgenic lines than in WT plants (Fig. 4f). After treatment, the relative expression of *AaBAM3.1* was higher in the treated transgenic plants than in the untreated transgenic plants (Fig. 4h). From the above results, we can infer the positive involvement of *AaBAM3.1* in enhancing the cold tolerance of kiwifruit.

### Promoter element analysis

We used the NewPLACE database and conducted a manual search to identify the cis-elements in the *AaBAM3.1* promoter sequences (~1.3 kb) that were involved in the plant cold response to reveal the transcriptional regulation network of the *AaBAM3.1* gene. A CBF binding site was identified in the promoter region of *AaBAM3.1*. ABRE, MYB, and ERF elements were also found in the promoter of *AaBAM3.1* (Table 1).

**Table 1:** *Cis*-acting regulatory elements were predicted in the promoter regions of *AaBAM3.1* in relation to the cold response of *A. arguta*

*Cis*-acting element name	Sequence	Probable function
CRT/DRE	TCGAC	CBF (C-repeat-binding factor)-binding
ABRE	ACGTC	Abscisic acid-responsiveness
MYB	CAACTG	
ERF	ATTTT/CAAA	
TGACG-motif	TGACG	MeJA-responsiveness
Box4	ATTAAT	Light responsiveness
MBS	CAACTG	Drought-inducibility
TCA-element	TCAGAAGAGG	Salicylic acid responsiveness
Circadian	CAAAGATATC	Circadian rhythm control

### Promoter activity assays for *AaBAM3.1*

**Fig. 5:**
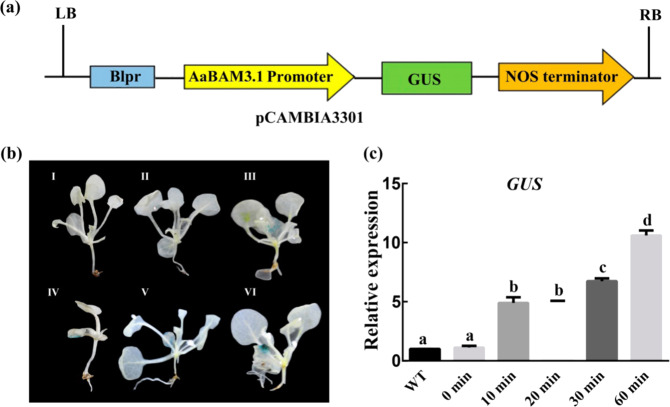
Analysis of *AaBAM3.1* promoter activity in transgenic *Arabidopsis* under cold stress. **a** Schematic diagram of PpCAMBIA3301-P_BAM_::GUS. **b** Analysis of GUS activity in transgenic *Arabidopsis* expressing the AaBAM3.1 promoter under cold stress (4 °C). I: WT; II: transgenic plant (4 °C for 0 min); III: transgenic plant (4 °C for 10 min); IV: transgenic plant (4 °C for 20 min); V: transgenic plant (4 °C for 30 min); VI: transgenic plant (4 °C for 60 min). **c** *GUS* mRNA expression was measured via RT-qPCR at 4 °C for 0 min, 10 min, 20 min, 30 min, and 60 min

For beta-glucuronidase (GUS) histological staining assays, the *AaBAM3.1* promoter sequence was ligated into a pCAMBIA3301 vector to obtain the recombinant plasmid *AaBAM3.1pro:GUS* (Fig. 5a). The *AaBAM3.1pro:GUS* gene in the transgenic plants was induced and overexpressed during cold stress. The relative expression of *GUS* showed that its activity gradually increased during cold stress treatment (Fig. 5b). Taken together, the results showed that the *AaBAM3.1* promoter played a key role in the response to cold stress.

### *AaCBF* expression during cold treatment and transcriptional activity assays

**Fig. 6:**
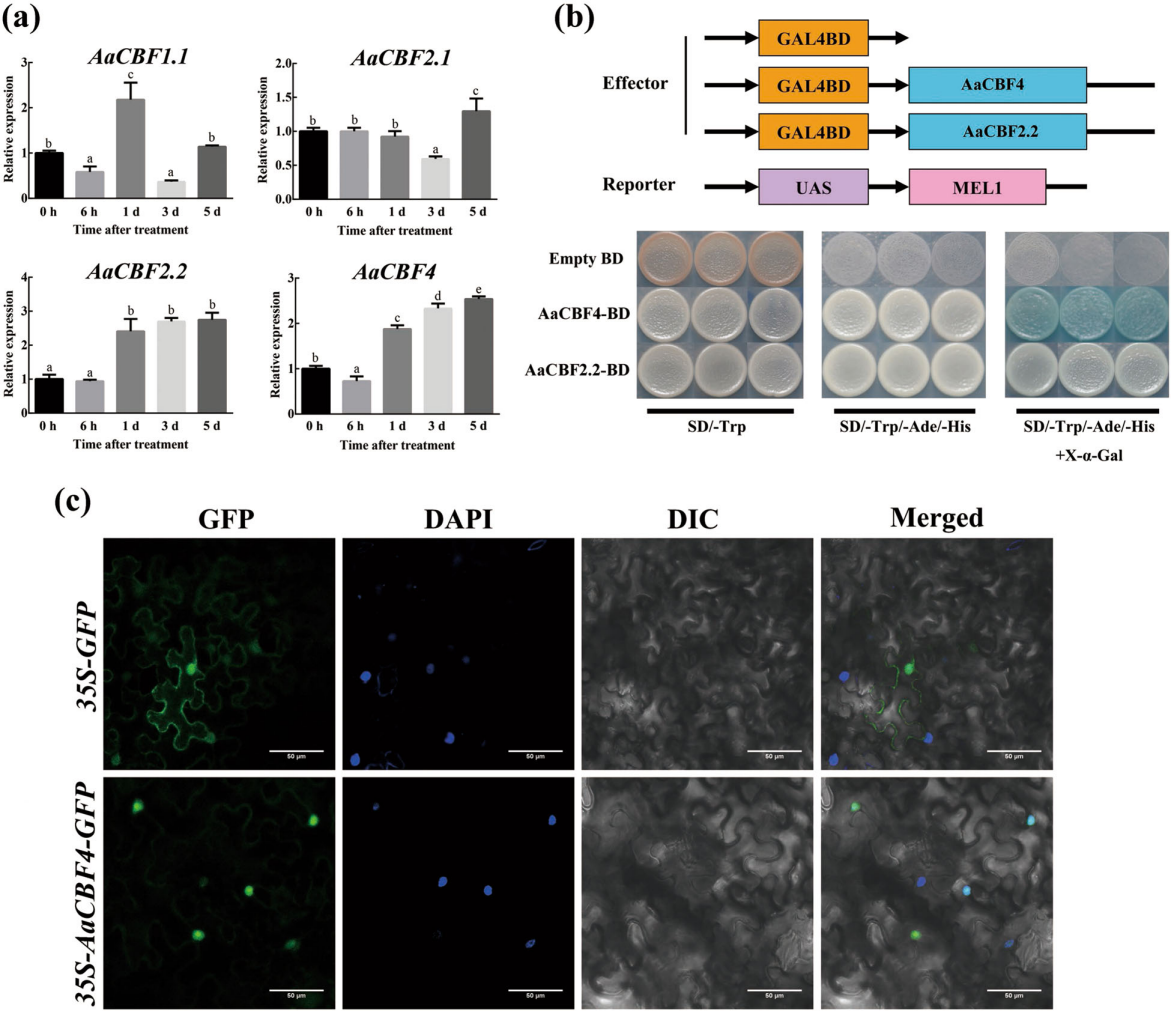
Analysis of *AaCBF4* sequence and protein characterization. **a** *AaCBF* expression was analyzed under cold stress. **b** Transactivation assay of CBFs in yeast. The fusion proteins of the GAL4 DNA-binding domain (BD) and full-length CBFs were expressed in yeast strain AH109. The empty vector pGBKT7 was used as a negative control. A culture solution of the transformed yeast was plated on SD/-Trp solid media, SD/-Trp/-Ade/-His solid media and SD/-Trp/-Ade/-His solid X-α-Gal solid media, as indicated. **c**. Subcellular localization of AaCBF4 in tobacco. Plant leaves were injected with *Agrobacterium tumefaciens* GV3101 containing the AaCBF4:eGFP vector. After 3 days, the leaves were observed for fluorescence with a confocal microscope (FV1000; Olympus, Tokyo, Japan). The nuclei were stained with DAPI

Four *AaCBF* genes were cloned and ligated into *A. arguta* using the homology-based cloning method; in accordance with the protocol for sequentially naming genes as they appear in the literature^44^, we named the four *AaCBF* genes *AaCBF1.1*, *AaCBF2.1*, *AaCBF2.2*, and *AaCBF4* (GenBank Accession Nos. MT477463, MT477464, MT477465, and MT477466). The trend of the mRNA transcript levels of *AaCBF2.2* and *AaCBF4* was the same as the expression of *AaBAM3.1* under cold treatment. In contrast, the expression of *AaCBF1.1* initially increased but then decreased with increasing treatment duration, and the *AaCBF2.1* transcript level remained low under cold treatment (Fig. 6a). Yeast strain AH109 containing pGBKT7-AaCBF2.2 and pGBKT7-AaCBF4 grew well on SD/-Trp/-Ade/-His plates, whereas only yeast cells transformed with pGBKT7-AaCBF4 exhibited GAL4 activity on SD/-Trp/-Ade/-His plates supplemented with X-α-Gal, indicating that AaCBF4 presented transcriptional activation activity (Fig. 6b). The results of our subcellular localization analysis are shown in Fig. 6c. The AaCBF4::GFP fusion protein was localized in the nuclear region of tobacco epidermal cells. Taken together, these results revealed that *AaCBF4* expression responded to cold exhibited and that this gene presented transcriptional activation activity.

### AaCBF4 binds to the *AaBAM3.1* promoter

**Fig. 7:**
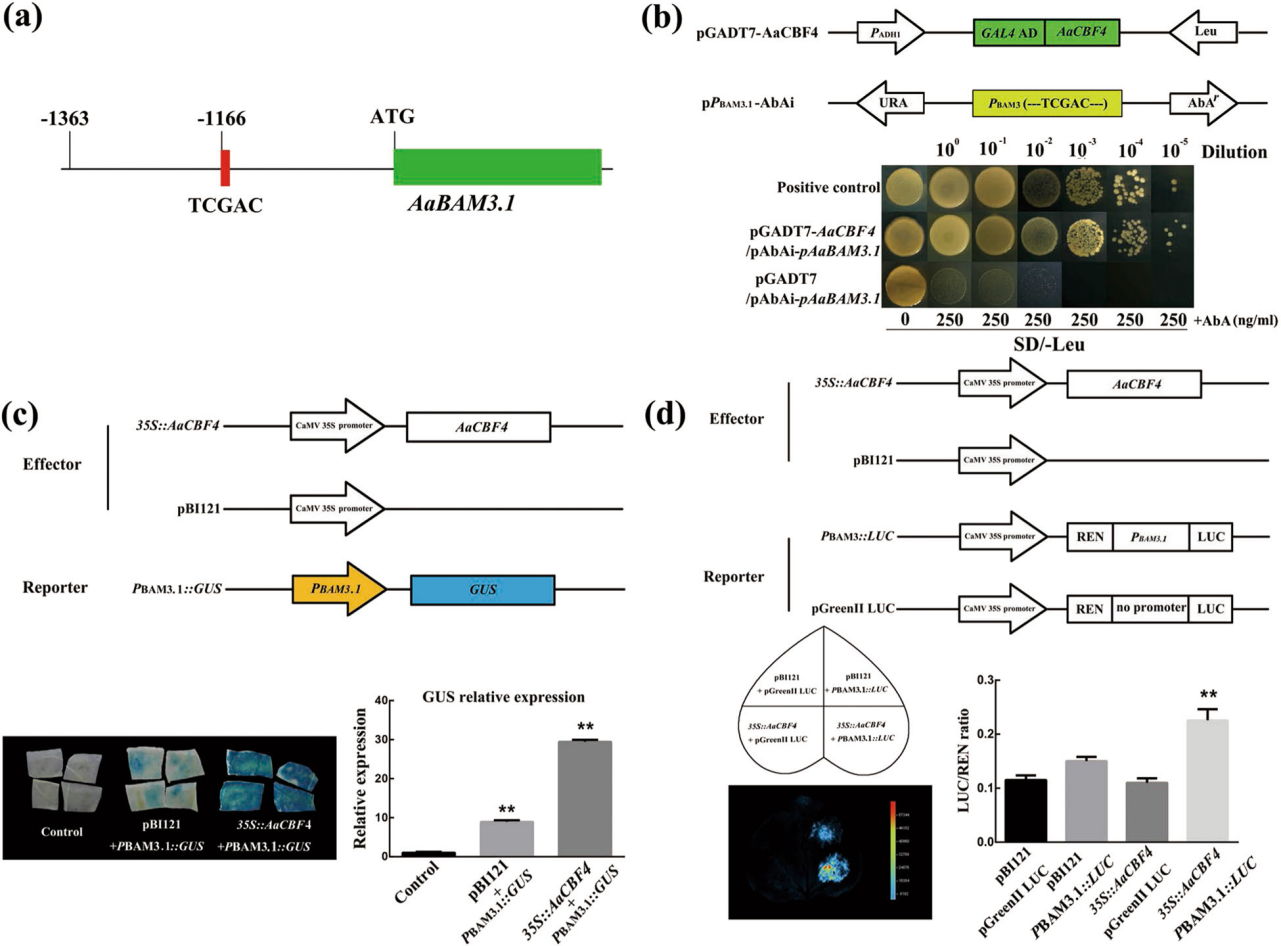
AaCBF4 binds to the CRT/DRE cis-element in the promoter of *AaBAM3.1*. **a** Structure of the *AaBAM3.1* promoter regions. A CRT/DRE cis-element was identified at the -1166 bp promoter position. **b** Yeast one-hybrid analysis using pGADT7-AaCBF4 as prey, pPBAM3.1-AbAi, and p-AbAi (empty vector) as bait and pGADT7-p53 and p53-AbAi as positive controls. **c** Transient glucuronidase (GUS) expression analysis using 35S::AaCBF4 as an effector and PBAM3.1::GUS as reporters. GUS expression was visualized in representative tobacco leaves transformed with different combinations of the effector and reporter constructs. **d** Luciferase activity analysis using 35S::AaCBF4 as the effector and 35S::LUC and PBAM3.1-35S::LUC as the reporters. REN and LUC activities resulting from different combinations of effector and reporter constructs were measured

Because the mRNA expression levels of *AaBAM*3.1 and *AaCBF4* increased in response to low temperature and because these genes play a key role in freezing tolerance, a *cis*-acting regulatory motif search of the *AaBAM3.1* promoter was performed to determine whether AaCBF4 could regulate the expression of *AaBAM3.1* under low temperature. We found a CBF-binding motif (TCGAC) located at −1166 bp in the *AaBAM3.1* promoter (Fig. 7a).

To verify the assumption that *AaBAM3.1* expression is directly regulated by AaCBF4, we performed a yeast one-hybrid assay (Y1H). The ORF of *AaCBF4* was cloned into the GAL4 activation domain in the pGADT7-Rec2 vector to construct the recombinant pGADT7-AaCBF4 plasmid (Fig. 7b). A 140 bp region of the *AaBAM3.1* promoter containing the CRT/DRE *cis*-element (TCGAC) was ligated and then cloned into a pAbAi vector to produce p*P*_*BAM3.1*_-AbAi bait (Fig. 7b). The pGADT7-AaCBF4 prey vector and p*P*_*BAM3.1*_-AbAi bait vectors were subsequently cotransformed into Y1H strains, and the transformed Y1H strains were then spread onto SD/-Leu media with or without 250 ng/mL AbA supplementation. pGADT7-p53 and p53-AbAi were used as positive controls. The pGADT7-AaCBF4 transformants and p*P*_*BAM3.1*_-AbAi transformants grew well on media supplemented with 250 ng/mL AbA, and the positive control (harboring pGADT7-p53 and p53-AbAi) also grew well on media supplemented with 250 ng/mL AbA, whereas the negative control (harboring pGADT7-AaCBF4 and empty p-AbAi) could not grow in the presence of 250 ng/mL AbA (Fig. 7b). These results indicated that AaCBF4 bound to the CRT/DRE *cis*-element in the promoter of *AaBAM3.1*.

To validate the results of the Y1H assays, we carried out a transient expression assay in tobacco leaves using a GUS activity assay. A 35 S::AaCBF4 fusion plasmid was constructed as an effector, and a recombinant P_BAM3.1_::GUS plasmid harboring 140 bp of the *AaBAM3.1* promoter sequence containing the CRT/DRE *cis*-element was used as a reporter. We observed that leaves cotransformed with 35 S::AaCBF4 and P_BAM3.1_::GUS exhibited a deeper blue color than did the leaves injected with the empty pBI121 effector and P_BAM3.1_::GUS; moreover, the relative GUS expression results were consistent with the GUS staining images (Fig. 7c).

We further performed a dual-luciferase reporter assay to confirm the Y1H results. The recombinant 35 S::AaCBF4 effector and two reporters, P_BAM3.1_::LUC and pGreenII::LUC, containing 140 bp AaBAM3.1 reporter sequences with or without the CRT/DRE cis-element, were constructed (Fig. 7d). The assays indicated that the LUC activities in the leaves infiltrated with 35 S::AaCBF4 and *P*_*BAM3.1*_::LUC were significantly higher than those in the leaves injected with 35 S::AaCBF4 and pGreenII::LUC or with the pBI121 empty vector and *P*_*BAM3.1*_::LUC **(**Fig. 7d**)**. The results of the LUC activity imaging were consistent with the LUC activity values.

## Discussion

### *AaBAM3.1* is expressed in response to cold

To survive cold stress, plants have developed the ability to resist low temperatures^45^. Many studies have revealed that plants degrade starch to produce soluble sugars and secondary metabolites to alleviate low-temperature injury^46^. Beta-amylase (mainly BAM3) is a major hydrolytic enzyme that attacks the nonreducing end of starch to release soluble sugars^47^. Among the nine *BAM* genes present in *A. thaliana*, only *AtBAM3* is transcriptionally induced by cold stress, which means that *AtBAM3* is a core gene involved in the mitigation of low-temperature injury in *A. thaliana*^48^. A previous study identified 16 *AcBAM* genes in the *A. chinensis* genome, and *AaBAM3.1*, obtained by homologous cloning, is a candidate gene for freezing tolerance in the dormant stage. According to the results of this study, *AaBAM3.1* can be categorized together with the cold-responsive *BAM3* genes of other species (*A. thaliana*, blueberry, orange, pear, tea) based on their sequence similarities and gene structure. In addition, BAM3 contains common domains that are conserved among different species. Based on these results, we can assume that starch metabolism is similar among plants in response to cold stress.

The activation of some BAM isoforms in response to cold stress has been reported according to gene expression and enzyme activity analyses. In rice, a *BMY8* mutant was found to be cold sensitive due to an inability to break down starch^33^. In our study, the photosynthesis system and membrane integrity were hindered by cold treatment, which upregulated *AaBAM3.1* mRNA transcript levels. We can assume that *AaBAM3.1* is as a prominent *BAM* gene involved in cold tolerance. First, AaBAM3.1 and AtBAM3 show high similarity in terms of domain conservation. Second, *AaBAM3.1* mRNA transcript levels continuously increased in response to low temperature over 5 days of treatment. In the growth period, the evaluation of the tissue-specific expression of this gene showed that the mRNA transcript level was highest in the leaves and lowest in the shoots. In the dormant period, the expression levels increased rapidly in the shoots. Taken together, these results showed that the plants adopted different strategies to cope with cold stress at different developmental stages in their annual growth cycle. Despite the different freezing tolerance strategies of the plants, *AaBAM3.1* expression was continuously induced under low temperature, which meant that *AaBAM3.1* played a role in the response to low-temperature stress and the enhancement of freezing tolerance at both the growth stage and the dormant stage.

### *A. arguta AaBAM3.1* may play a role in enhancing freezing tolerance

Cold stress is widely known to induce osmotic stress, which can trigger a decrease in membrane ion exchange, metabolic disorders, a loss of water potential and cell death, depending on the duration and degree of low-temperature treatment^49^. Plants have developed sophisticated strategies for adapting to or surviving under cold stress, and one of these survival strategies involves the accumulation of very high levels of osmolytes or compatible solutes through osmotic adjustment^50^. Sugars are considered important osmolytes that can help plant cells resist abiotic stresses, improve water retention, regulate and stabilize biochemical reactions and protect membranes by adjusting the osmotic potential within the cell^51^. Sugar metabolism involves many pathways that produce large quantities of soluble sugars, such as sucrose, glucose, fructose, trehalose, fructans, and raffinose^52^. Plants treated with exogenous soluble sugars before cold stress have been shown to exhibit high accumulations of endogenous soluble sugars, showing that these metabolites and their derived products can act as compatible solutes that play key roles in protecting plant cells from injury induced by cold stress^53^. A positive correlation between the mRNA expression level of BAM3 and soluble sugar accumulation has been observed under cold stress, which leads to enhanced freezing tolerance^54^. Compared with WT plants, *AtBAM3* RNAi lines show decreased soluble sugar levels and poor freezing tolerance under cold stress, indicating that these characteristics might be positively correlated. In this study, the higher accumulation of compatible solutes observed in *AaBAM3.1*-overexpressing transgenic lines (both *A. thaliana* and kiwifruit plants) led us to speculate that the transgenic plants overexpressing *AaBAM3.1* may have a stronger osmotic adjustment ability than WT plants have. This assumption was supported by the low level of REL observed under low temperature, indicating that, compared with the WT plants, the *AaBAM3.1-*overexpressing lines experienced minor osmotic stress injury. Consequently, the higher accumulation of soluble sugars was demonstrated to be one of the survival mechanisms enhancing freezing tolerance in the *AaBAM3.1-*overexpressing transgenic lines compared with the WT plants.

Cold stress disrupts the redox balance in cells, which leads to the accumulation of additional ROS^55^. In turn, high ROS accumulation leads to oxidative stress, which is harmful to cellular functions and biological processes because of the denaturation of functional proteins^56^. Under nonstress conditions, the amount of ROS production is equal to that of ROS scavenging, while increased ROS accumulation is mainly due to an imbalance between ROS production and scavenging under cold stress. To survive oxidative stress, plants have evolved complex strategies to scavenge various types of ROS. Some of the well-known antioxidant compounds can be divided into two categories: enzymatic (SOD, POD, CAT, etc.) and nonenzymatic (anthocyanin, carotenoids, glutathione, tocopherols and ascorbic acid, etc.) ones^57–60^. Recent findings have shown that several sugars can serve as antioxidants and have an important effect on ROS scavenging, including glucose, fructose, sucrose, inositol, galactinol, trehalose and raffinose^18^. It is clear that the regulation of the plant redox system can increase freezing tolerance^61^. In this study, DAB and NBT staining assays indicated that the accumulation of antioxidants in *AaBAM3.1*-overexpressing plants was greater than that in WT plants, suggesting that the *AaBAM3.1*-overexpressing plants present a robust ability to scavenge ROS under cold stress. Moreover, the lower accumulation of MDA, which serves as an indicator of the degree of membrane damage, showed that the *AaBAM3.1*-overexpressing transgenic lines experienced minor oxidative stress. Chloroplasts and mitochondria are the major organelles responsible for ROS generation due to the disorder of the electron transfer chain. Interestingly, subcellular localization results showed that AaBAM3.1 localizes to the chloroplast, implying that the AaBAM3.1 enzyme in chloroplasts might be directly and indirectly involved in scavenging ROS. Soluble sugars derived from starch degradation in chloroplasts can play a key role in sugar metabolism to produce various sugar derivatives through a set of sugar metabolism pathways. Therefore, we assume that, compared with the WT plants, the overexpressing lines accumulated greater amounts of soluble sugars and their derivatives to scavenge the ROS produced in the chloroplasts. ROS produced in the cytoplasm can also be scavenged by soluble sugars^62^. Overall, the results revealed that the increased ROS-scavenging ability derived from the highly abundant accumulation of soluble sugars is a survival mechanism that enhances the freezing tolerance of kiwifruit.

### A model for the regulation of freezing tolerance driven by the *AaCBF4-AaBAM3.1* module

The *BAM* gene is a functional gene encoding an enzyme with catalytic activity. Moreover, it plays a direct role in alleviating cold damage by producing soluble sugars contributing to cold stress resistance. However, the specific role of the *BAM* gene in transcriptional regulatory networks is poorly understood. The OsMYB30 transcription factor can suppress the expression of *BMY2*, *BMY6*, and *BMY10* in rice^63^. In the past few decades, the CBF regulatory pathway has become the best-understood cold-responsive network^64^. It has been shown that CBFs act as a hub linking the downstream low-temperature response with upstream signal transmission. In some species, including *A. thaliana*, *Triticum aestivum*, *Hordeum vulgare*, and *Brassica oleracea*, some *CBF* genes have been shown to serve as indicators of freezing tolerance. The expression of a number of COR genes is regulated by CBFs, which bind to corresponding CRT/DRE *cis-*elements in the promoters of the COR genes. The genes containing CRT/DRE *cis-*elements in their promoters encode a series of different cryoprotectant proteins, including COR15a, ERD10, COR6.6, KIN1, RD19a, and COR47, which play key roles in enhancing freezing tolerance^65^. However, although some COR genes containing CRT/DRE *cis-*elements in their promoters have been identified, the knowledge of the genes regulated by CBFs and their functions in freezing tolerance of kiwifruit is still quite poor. In this study, we found that the *AaCBF4* gene could respond to cold stress and tended to be expressed in the same manner as the *AaBAM3.1* gene was under cold stress. The *AaCBF4* gene encodes a TF involved in the positive regulation of the freezing tolerance of kiwifruit. A CBF-binding element has been identified in the *AaBAM3.1* promoter region. Y1H, transient *GUS* and dual-luciferase reporter assays indicated that AaCBF4 showed a positive relationship with *AaBAM3.1*. On the basis of these results, we speculated that *AaBAM3.1* expression is regulated by AaCBF4, indicating that the *AaCBF*-*AaBAM* module may be activated by cold stress to enhance freezing tolerance.

## Conclusion

In conclusion, our results demonstrate that *AaBAM3.1*, which is localized in the chloroplasts, is responsible for freezing tolerance enhancement and is involved in starch degradation. *AaBAM3.1* plays a key role in enhancing freezing tolerance through the modulation of compatible solute and antioxidant enzyme levels. *AaBAM3.1* is regarded as a cold-responsive gene containing CRT/DRE elements in its promoters. Hence, these findings show that the *AaCBF4-AaBAM3.1* module is involved in the transduction network related to the freezing tolerance of kiwifruit.

## Materials and methods

### Plant materials and treatments

Two *Actinidia* species, *A. arguta* cv. Kuilv (KL) (whose semilethal temperature is −30 °C) and *A. chinensis* cv. Hongyang (whose semilethal temperature is −13 °C) were used in this study. Micropropagated plantlets were grown on Murashige and Skoog 1962 (MS) culture media comprising 7.2 g/L agar and 30 g/L sucrose (pH 5.8). Thirty-day-old plants displaying uniform growth were used for cold treatments. Plants grown in bottles were treated at 4 °C in a low-temperature incubator, and whole leaves were then harvested at 0 h, 6 h, 1 d, 3 d, and 5 d time points. Each sample included three biological replicates. For seasonal gene expression samples, mature (3-year-old) KL plants planted in 3-l pots were placed in the field. The shoots were collected from mid-April 2017 to March 2018. All six of the collection points were in the morning: S1, 15 April; S2, 15 November; S3, 7 December; S4, 23 December; S5, 15 January; and S6, 15 March. Moreover, different tissues of KL plants were collected. Three biological replicates of shoots were used for RNA extraction.

### Gene cloning and sequence analysis

The open reading frames (ORFs) of *AaBAM3.1* and *AaCBF4* were cloned from the cDNA of *A. arguta* (KL) leaves by using the primers AaBAM3.1-ORF-F/R and AaCBF4-ORF-F/R (Table S1). AaCBF1.1, AaCBF2.1 and AaCBF2.2 were cloned by using the primers AaCBF1.1-ORF-F/R, AaCBF2.1-ORF-F/R and AaCBF2.2-ORF-F/R, respectively. Multiple alignment of the putative protein sequences was conducted via DNAMAN 7.0. MEGAX 32 software was utilized to construct a maximum likelihood (ML) tree with 1000 bootstrap replicates. Tools from the online website ExPASy (http://www.expasy.org) were used to predict the molecular weights and theoretical isoelectric points. Furthermore, the ClustalX program with default settings was employed to predict the conserved domains. Underlying *cis*-elements in the promoter sequence were identified via the NewPLACE (https://www.dna.affrc.go.jp/PLACE/) database.

### Quantitative real-time PCR (RT-qPCR) analysis

The RNA extraction method and RT-qPCR were performed as described by Li^66^. Three independent biological replicates were analyzed for each sample. All the data were analyzed using the 2^−△△Ct^ method^67^. The RT-qPCR primers were designed by using Primer Premier 5 (the primers used are listed in Table S1).

### Subcellular localization of AaBAM3.1 and AaCBF4

The coding sequences of *AaBAM3.1* and *AaCBF4* were amplified via HiFi-enzyme PCR using primers without stop codons. The PCR products of *AaBAM3.1* and *AaCBF4* were then ligated into a pBI121 plasmid and fused together with GFP, and their expression was driven by the CaMV 35 S promoter. Following sequence confirmation, the recombinant 35 S pro:AaBAM3.1::GFP, 35 S pro:AaCBF4::GFP, and 35 S pro:GFP (control) plasmids were transferred into *Agrobacterium tumefaciens* strain GV3101. Tobacco leaves were injected with these bacterial suspensions (OD_600_ = 0.6–1.0), which were then kept in a chamber for 2–3 days, after which the transformed leaves were then observed under a confocal laser-scanning microscope (FV1000; Olympus, Tokyo, Japan).

### Detection of BAM enzyme activity

The *AaBAM3.1* cDNA was cloned by using primers without stop codons. The PCR product was subcloned into pGEX4T-1 with a GST tag. The fusion plasmid and EV were subsequently transferred into the *E*. *coli* Rosetta (DE3) strain. *E*. *coli* cells harboring pGEX4T-1-AaBAM3.1 and pGEX4T-1 (EV) were then cultivated in 50 mL of liquid LB media consisting of 50 mg/L kanamycin at 37 °C to an OD_600_ = 0.6 and then induced with 1 mM isopropyl-beta-D-thiogalactopyranoside (IPTG) at 28 °C. After 8 h, the cells were collected by centrifugation at 10,000 × g for 10 min, resuspended in phosphate-buffered saline (PBS; pH = 7.0), and disrupted by sonication. Enzyme activity was measured using a beta-amylase assay kit (Nanjing Jiancheng Bioengineering Institute, China).

### Y1H assays

Y1H assays were conducted using a Matchmaker^TM^ Gold Yeast One-Hybrid Library Screening System (Clontech, San Francisco, USA) to examine the interaction of AaCBF4 and the *AaBAM3.1* promoter. The primers listed in Table S1 were used to clone the 140 bp promoter region containing a CRT/DRE *cis*-element. The amplified 140 bp sequence was cloned into an pAbAi vector to construct bait. The *AaCBF4* ORF was fused with the GAL4 activation domain (AD) in the pGADT7-AD vector to produce a prey vector (pGAD-AaCBF4). After the transformants were screened on SD/-Ura plates and the minimal inhibitory concentration of aureobasidin A (AbA) for the positive bait strains was measured, pGAD-AaCBF4 was transferred to yeast cells. A positive yeast strain was selected on SD/-Leu plates that included 250 ng/mL AbA and were cultured at 30 °C for 2–3 days. Positive yeast cells harboring pGADT7-53 and p53-AbAi were used as positive controls.

### GUS and luciferase reporter assays

To verify the interaction between AaCBF4 and the CRT/DRE cis-elements in the *AaBAM3.1* promoter, transient expression experiments and GUS and luciferase reporter assays were conducted in the model plant species tobacco^68^. The PCR-amplified 140 bp promoter sequence containing the *AaBAM3.1* promoter CRT/DRE *cis*-element was cloned into pBI121 and pGreenII 0800-LUC vectors to obtain reporter plasmids. The *AaCBF4* ORF was ligated into pBI121 to produce effector constructs. The effector and reporter constructs were subsequently transferred into *A. tumefaciens* strain GV3101, which were then coinfiltrated into tobacco leaves via agroinfiltration^69^. After coculturing in an illuminated chamber for 48–72 h at 25 °C, a Dual-Luciferase Reporter Assay System (Promega, USA) was used to determine LUC and REN luciferase activities according to the manufacturer’s instructions, and GUS staining was performed as described by Hellens^70^.

### *A. thaliana* transformation

The *AaBAM3.1* ORF driven by the constitutive CaMV 35 S promoter or *AaBAM3.1* promoter was cloned into pBI121 or pCAMBIA3301. *A. tumefaciens* (strain GV3101) harboring the *AaBAM3.1* ORF or *AaBAM3.1* promoter-containing recombinant plasmids were transformed into WT *A. thaliana* (Col-0) plants via the floral-dip method^71^. T0 seeds were selected on MS screening culture media comprising kanamycin (Kan) (100 mg/L; Sigma, St. Louis, USA) or phosphinothricin (Basta) (1 mg/L; Sigma, St. Louis, USA). Three positive T3 transgenic lines were chosen for further gene functional verification.

### Cold treatment of transgenic *A. thaliana* and analysis of physiological characteristics

For the freezing tolerance experiment, 3-week-old WT and transgenic *A. thaliana* plants (Fig. S1) were treated as described by Ding^72^. The survival was scored 7 days after treatment. For the determination of physiological changes, 3-week-old *A. thaliana* plants from the WT and transgenic lines were subjected to treatment at −2 °C for 2 h. Afterward, SOD, POD, CAT, and BAM activities and PRO, SS and MDA contents were determined as described in the kit manual (Nanjing Jiancheng Bioengineering Institute, China). REL was measured via the method described by Lappi^73^. Using the 3,3’-diaminobenzidine (DAB) and nitro blue tetrazolium (NBT) staining methods, the accumulation of hydrogen peroxide (H_2_O_2_) and superoxide (O_2_^−^) under cold stress was determined^74,75^. For chlorophyll fluorescence measurements, whole plants were imaged using an IMAGING-PAM chlorophyll fluorometer (Walz, Effeltrich, Germany), and plants were placed in a dark room for 20 min prior to imaging. The maximum quantum efficiency of photosystem II (*Fv/Fm*) was measured with Imaging WinGegE software^76^. For GUS staining, 3-week-old *A. thaliana* plants (Fig. S3) from the WT and transgenic lines were subjected to 4 °C for 0 min, 10 min, 20 min, 30 min, or 60 min. Each sample consisted of three seedlings, and each experiment was performed in triplicate.

### Transformation of *A. chinensis* leaves and regeneration of transgenic plants

The *AaBAM3.1* ORF was cloned into a pBI121 vector driven by the CaMV 35 S promoter, and the recombinant plasmid was subsequently transformed into *A. chinensis* leaves based on the protocol outlined by Wang^77^ **(**Fig. S2**)**. Transgenic plants were obtained after ~6 months. The transformed plants were identified using PCR and RT-qPCR methods to successfully verify the incorporation of the transgene. Three positive transgenic kiwifruit lines with high *AaBAM3.1* mRNA expression were chosen for molecular and phenotypic analyses.

### Evaluation of the cold tolerance of *AaBAM3.1-*overexpressing *A. chinensis* plants

*A. chinensis* plants from the WT and three transgenic lines (OE#1, OE#2, OE#3) were subjected to cold stress (at 2 °C) for 2 h. After cold treatment, plant leaves were harvested to measure various physiological indicators. The BAM activity and SS contents were determined based on the abovementioned methods. The REL and MDA contents were measured according the abovementioned methods. The in situ accumulation of H_2_O_2_ and O_2_^−^ was detected via DAB and NBT, respectively. For chlorophyll fluorescence detection, whole plants were imaged by an IMAGING-PAM chlorophyll fluorometer, and the maximum quantum efficiency of photosystem II (*Fv/Fm*) was determined by Imaging WinGegE software.

### Statistical analysis

The statistical analysis was performed with the Windows-based SPSS software package (version 22, USA). T-tests and one-way ANOVA were used to analyze the data, and Duncan’s multiple comparisons were employed for sample comparisons at significance levels of *P* < 0.05 or *P* < 0.01.

## Supplementary information

supplemental figure

supplemental table

## References

[1] Kou S (2018). The arginine decarboxylase gene ADC1, associated to the putrescine pathway, plays an important role in potato cold-acclimated freezing tolerance as revealed by transcriptome and metabolome analyses. Plant J..

[2] Stockinger EJ, Gilmour SJ, Thomashow MF (1997). Arabidopsis thaliana CBF1 encodes an AP2 domain-containing transcriptional activator that binds to the C-repeat/DRE, a cis-acting DNA regulatory element that stimulates transcription in response to low temperature and water deficit. Proc. Natl Acad. Sci. USA.

[3] Wisniewski M, Bassett C, Gusta LV (2003). An overview of cold hardiness in Woody plants: Seeing the forest through the trees. Hortscience.

[4] Wisniewski M, Nassuth A, Arora R (2018). Cold hardiness in trees: a mini-review. Front. Plant Sci..

[5] Chen MJ (2020). Characterization of low temperature-induced plasma membrane lipidome remodeling combined with gene expression analysis reveals mechanisms that regulate membrane lipid desaturation in Carica papaya. Sci. Hortic..

[6] Ma Y (2015). COLD1 confers chilling tolerance in rice. Cell.

[7] Zhao CZ (2017). MAP kinase cascades regulate the cold response by modulating ICE1 protein stability. Dev. Cell.

[8] He YA (2016). Phytochrome B negatively affects cold tolerance by regulating OsDREB1 gene expression through phytochrome interacting factor-like protein OsPIL16 in rice. Front. Plant Sci..

[9] Li H (2017). BZR1 positively regulates freezing tolerance via CBF-dependent and CBF-independent pathways in arabidopsis. Mol. Plant..

[10] Hu HH, Xiong LZ (2014). Genetic engineering and breeding of drought-resistant crops. Annu. Rev. Plant Biol..

[11] Dreyer A, Dietz KJ (2018). Reactive oxygen species and the redox-regulatory network in cold stress acclimation. Antioxid.-basel.

[12] Chu MX (2018). AtCaM4 interacts with a Sec14-like protein, PATL1, to regulate freezing tolerance in Arabidopsis in a CBF-independent manner. J. Exp. Bot..

[13] Ishikawa, M. et al. Ice Nucleation Activity in Plants: The Distribution, Characterization, and Their Roles in Cold Hardiness Mechanisms in Survival Strategies in Extreme Cold and Desiccation: Adaptation Mechanisms and Their Applications Vol. 1081 (eds IwayaInoue, M., Sakurai, M. & Uemura, M.) 99-115 (Springer-Verlag Singapore Pte Ltd, 2018). 10.1007/978-981-13-1244-1_6 30288706

[14] Karami H, Rezaei M, Sarkhosh A, Rahemi M, Jafari M (2018). Cold hardiness assessment in seven commercial fig cultivars (Ficus CaricaL.). Gesund. Pflanz..

[15] Zhang H, Wang YP, Wang WE, Bao MZ, Chan ZL (2019). Physiological changes and DREB1s expression profiles of tall fescue in response to freezing stress. Sci. Hortic..

[16] Miller G, Suzuki N, Ciftci-Yilmaz S, Mittler R (2010). Reactive oxygen species homeostasis and signalling during drought and salinity stresses. Plant Cell Environ..

[17] Cabello JV, Lodeyro AF, Zurbriggen MD (2014). Novel perspectives for the engineering of abiotic stress tolerance in plants. Curr. Opin. Biotechnol..

[18] Keunen E, Peshev D, Vangronsveld J, Van den Ende W, Cuypers A (2013). Plant sugars are crucial players in the oxidative challenge during abiotic stress: extending the traditional concept. Plant Cell Environ..

[19] Dietz KJ (2008). Redox signal integration: from stimulus to networks and genes. Physiol. Plant..

[20] Adams CA, Rinne RW (1981). The occurrence and significance of dispensable proteins in plants. N. Phytol..

[21] Hildebrand DF, Hymowitz T (1980). The Sp1 locus in soybean codes for beta -amylase. Crop Sci..

[22] Thalmann M, Santelia D (2017). Starch as a determinant of plant fitness under abiotic stress. N. Phytol..

[23] Sun SH (2020). Freezing tolerance and expression of beta-amylase gene in two actinidia arguta cultivars with seasonal changes. Plants-Basel.

[24] Fulton DC (2008). β-AMYLASE4, a noncatalytic protein required for starch breakdown, acts upstream of three active β-amylases in arabidopsis chloroplasts. Plant Cell.

[25] Zeeman SC (2004). Plastidial alpha-glucan phosphorylase is not required for starch degradation in Arabidopsis leaves but has a role in the tolerance of abiotic stress. Plant physiol..

[26] Hyung LJ, Jun YD, Jin KS, Doil C, Jae LH (2012). Intraspecies differences in cold hardiness, carbohydrate content and β-amylase gene expression of Vaccinium corymbosum during cold acclimation and deacclimation. Tree Physiol..

[27] Hao XY, Yue C, Tang H, Qian WJ, Yang YJ (2017). Cloning of β-amylase Gene (CsBAM3) and Its Expression Model Response to Cold Stress in Tea Plant. Acta Agronomica Sin..

[28] Peng T, Zhu X, Duan N, Liu JH (2015). PtrBAM1, a β-amylase-coding gene of Poncirus trifoliata, is a CBF regulon member with function in cold tolerance by modulating soluble sugar levels. Plant Cell Environ..

[29] Liangyi (2018). Transcriptomic and evolutionary analyses of white pear (Pyrus bretschneideri) β-amylase genes reveals their importance for cold and drought stress responses. Gene.

[30] Nielsen TH, Deiting U, Stitt M (1997). A beta-amylase in potato tubers is induced by storage at low temperature. Plant physiol..

[31] Dong SY, Beckles DM (2019). Dynamic changes in the starch-sugar interconversion within plant source and sink tissues promote a better abiotic stress response. J. Plant Physiol..

[32] Monroe JD, Storm AR (2018). Review: the arabidopsis β-amylase (BAM) gene family: diversity of form and function. Plant Sci..

[33] Kaplan F, Guy CL (2010). RNA interference of Arabidopsis beta-amylase8 prevents maltose accumulation upon cold shock and increases sensitivity of PSII photochemical efficiency to freezing stress. Plant J..

[34] Hou J (2017). Amylases StAmy23, StBAM1 and StBAM9 regulate cold-induced sweetening of potato tubers in distinct ways. J. Exp. Bot..

[35] An JP (2017). MdHY5 positively regulates cold tolerance via CBF-dependent and CBF-independent pathways in apple. J. Plant Physiol..

[36] Lee (2015). The unified ICE-CBF pathway provides a transcriptional feedback control of freezing tolerance during cold acclimation in Arabidopsis. Plant Mol. Biol..

[37] Cook D, Fowler S, Fiehn O, Thomashow MF (2004). A prominent role for the CBF cold response pathway in configuring the low-temperature metabolome of Arabidopsis. Proc. Natl Acad. Sci. USA.

[38] Feng WQ, Li J, Long SX, Wei SJ (2019). A DREB1 gene from zoysiagrass enhances Arabidopsis tolerance to temperature stresses without growth inhibition. Plant Sci..

[39] Mizoi J, Shinozaki K, Yamaguchi-Shinozaki K (2012). AP2/ERF family transcription factors in plant abiotic stress responses. Biochim. Biophys. Acta-Gene Regul. Mech..

[40] Cho S (2017). Accession-dependent CBF gene deletion by CRISPR/Cas system in Arabidopsis. Front. Plant Sci..

[41] Liu YF (2017). Rapid radiations of both kiwifruit hybrid lineages and their parents shed light on a two-layer mode of species diversification. N. Phytol..

[42] Sun S, Qi X, Wang R, Lin M, Fang J (2020). Evaluation of freezing tolerance in Actinidia germplasm based on relative electrolyte leakage. Hortic. Environ. Biote..

[43] Jo, Y. S., Cho, H. S., Park, J. O., Kim, T. C. & Kim, W. S. Assay of Cold Tolerance of Actinidia eriantha in Proceedings of the International Symposium on Citrus and Other Tropical and Subtropical Fruit Crops Vol. 41 (ed Oh, D. G.) 277-282 (Int Soc Horticultural Science, 2008).

[44] Gunaseelan K (2019). Copy number variants in kiwifruit ETHYLENE RESPONSE FACTOR/APETALA2 (ERF/AP2)-like genes show divergence in fruit ripening associated cold and ethylene responses in C-REPEAT/DRE BINDING FACTOR-like genes. PLOS ONE.

[45] An JP (2018). R2R3-MYB transcription factor MdMYB23 is involved in the cold tolerance and proanthocyanidin accumulation in apple. Plant J..

[46] Zhang AD (2018). Transcriptome Analysis Identifies a Zinc Finger Protein Regulating Starch Degradation in Kiwifruit. Plant physiol..

[47] Zeeman SC, Kossmann J, Smith AM (2010). Starch: Its Metabolism, Evolution, and Biotechnological Modification in Plants. Annu. Rev. Plant Biol..

[48] Nakaminami K (2014). Analysis of Differential Expression Patterns of mRNA and Protein During Cold-acclimation and De-acclimation in Arabidopsis. Mol. Cell. Proteom..

[49] Zhao Y (2020). Analysis of the cold tolerance and physiological response differences of amur grape (Vitis amurensis) germplasms during overwintering. Sci. Hortic..

[50] Kaya O, Kose C (2017). Determination of resistance to low temperatures of winter buds on lateral shoot present in Karaerik (Vitis vinifera L.) grape cultivar. Acta Physiol. Plant..

[51] Concetta (2010). Thioredoxin-regulated β-amylase (BAM1) triggers diurnal starch degradation in guard cells, and in mesophyll cells under osmotic stress. J. Exp. Bot..

[52] Yue C (2015). Effects of cold acclimation on sugar metabolism and sugar-related gene expression in tea plant during the winter season. Plant Mol. Biol..

[53] Uemura M, Steponkus PL (2010). Modification of the intracellular sugar content alters the incidence of freeze-induced membrane lesions of protoplasts isolated from Arabidopsis thaliana leaves. Plant Cell Environ..

[54] Kaplan F, Guy CL (2004). beta-amylase induction and the protective role of maltose during temperature shock. Plant physiol..

[55] Moon JC (2015). Overexpression of Arabidopsis NADPH-dependent thioredoxin reductase C (AtNTRC) confers freezing and cold shock tolerance to plants. Biochem. Bioph. Res. Co..

[56] Huang XS, Wang W, Zhang Q, Liu JH (2013). A basic helix-loop-helix transcription factor, PtrbHLH, of poncirus trifoliata confers cold tolerance and modulates peroxidase-mediated scavenging of hydrogen peroxide. Plant physiol..

[57] Asada, Kozi (1999). The water-water cycle in chloroplasts: scavenging of active oxygens and dissipation of excess photons. Annu Rev. Plant Physiol. Plant Mol. Biol..

[58] Foyer CH, Shigeoka S (2011). Understanding oxidative stress and antioxidant functions to enhance photosynthesis. Plant physiol..

[59] Wang X (2013). Differential antioxidant responses to cold stress in cell suspension cultures of two subspecies of rice. Plant Cell Tiss. Org..

[60] Dietz, Karl-Josef (2011). Peroxiredoxins in plants and cyanobacteria. Antioxid. Redox Sign..

[61] Amrina S (2014). Simultaneous over-expression of PaSOD and RaAPX in transgenic Arabidopsis thaliana confers cold stress tolerance through increase in vascular lignifications. Plos One.

[62] Wei T (2019). Enhanced ROS scavenging and sugar accumulation contribute to drought tolerance of naturally occurring autotetraploids in Poncirus trifoliata. Plant Biotechnol. J..

[63] Lv Y (2017). The OsMYB30 transcription factor suppresses cold tolerance by interacting with a JAZ protein and suppressing beta-amylase expression. Plant physiol..

[64] Dong XJ (2020). The cold response regulator CBF1 promotes Arabidopsis hypocotyl growth at ambient temperatures. Embo J..

[65] Townley HE, Knight MR (2002). Calmodulin as a potential negative regulator of Arabidopsis COR gene expression. Plant Physiol..

[66] Li YK (2018). Combined analysis of the fruit metabolome and transcriptome reveals candidate genes involved in flavonoid biosynthesis in actinidia arguta. Int. J. Mol. Sci..

[67] Livak KJ, Schmittgen TD (2001). Analysis of relative gene expression data using real-time quantitative PCR and the 2(T)(-Delta Delta C) method. Methods.

[68] Wang XB (2019). PpERF3 positively regulates ABA biosynthesis by activating PpNCED2/3 transcription during fruit ripening in peach. Hortic. Res..

[69] Jefferson RA, Kavanagh TA, Bevan MW (1987). GUS fusion: beta-glucuronidase as a sensitive and versatile gene fusion marker in higher plants. Embo J..

[70] Hellens RP (2005). Transient expression vectors for functional genomics, quantification of promoter activity and RNA silencing in plants. Plant Methods.

[71] Davis A, Hall A, Millar A, Darrah C, Davis S (2009). Protocol: Streamlined sub-protocols for floral-DIP transformation and selection of transformants in Arabidopsis thaliana. Plant Methods.

[72] Ding YL (2019). EGR2 phosphatase regulates OST1 kinase activity and freezing tolerance in Arabidopsis. Embo J..

[73] Lappi J, Luoranen J (2018). Testing the differences of LT 50, LD 50, or ED 50. Can. J. For. Res..

[74] ThordalChristensen H, Zhang ZG, Wei YD, Collinge DB (1997). Subcellular localization of H2O2 in plants. H2O2 accumulation in papillae and hypersensitive response during the barley-powdery mildew interaction. Plant J..

[75] Jabs T, Dietrich RA, Dangl JL (1996). Initiation of runaway cell death in an Arabidopsis mutant by extracellular superoxide. Science.

[76] Su LY, Dai ZW, Li SH, Xin HP (2015). A novel system for evaluating drought-cold tolerance of grapevines using chlorophyll fluorescence. BMC Plant Biol..

[77] Wang T, Atkinson R, Janssen B (2007). The choice of agrobacterium strain for transformation of kiwifruit. Acta Horticulturae.

