# Supplementary material for: The AaCBF4-AaBAM3.1 module enhances freezing tolerance of kiwifruit (Actinidia arguta)
Source: Hortic Res. 2021 May 1;8:97. doi: 10.1038/s41438-021-00530-1 (PMC8087828; doi:10.1038/s41438-021-00530-1)
Supplement: Supplementary file 1 — supplemental figure [file 41438_2021_530_MOESM1_ESM.docx]

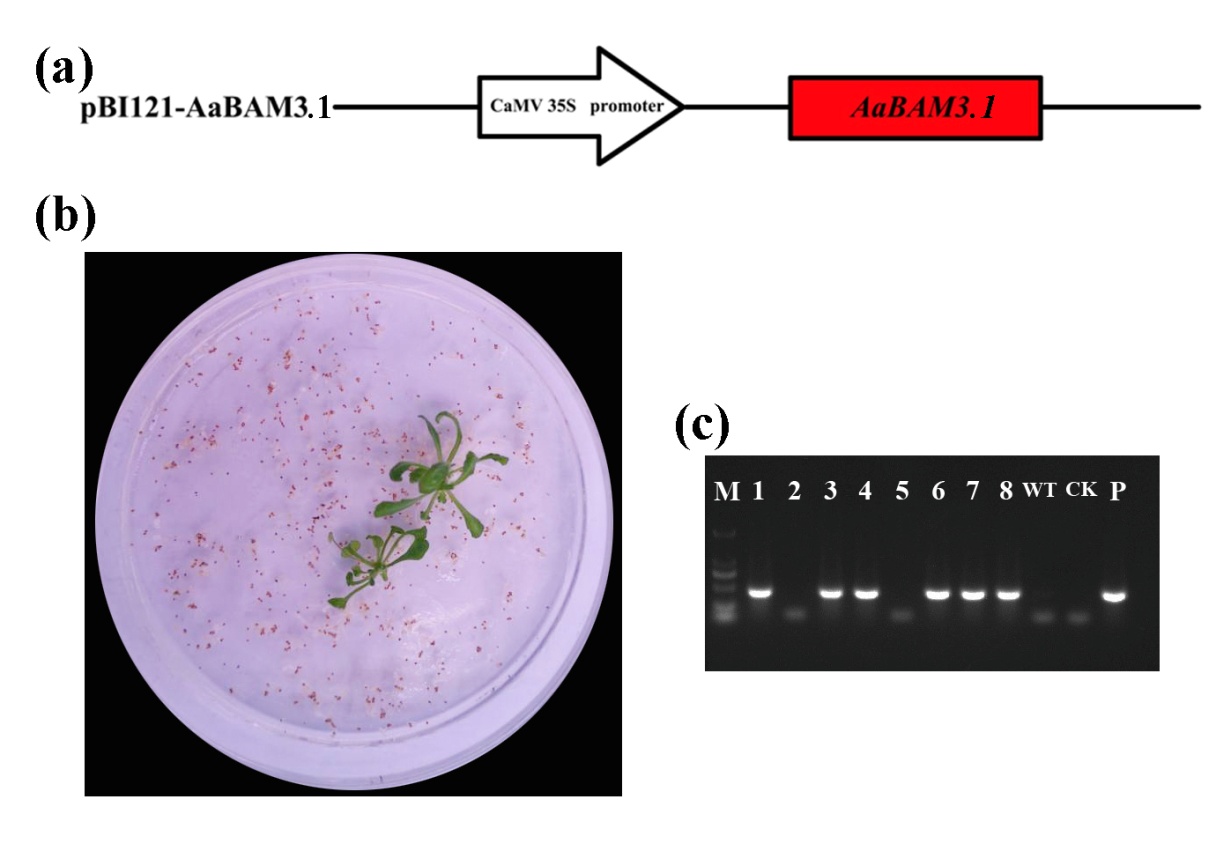


Figure S1. Positive trangenic screening of overexpressed *AaBAM3.1* lines in *A. thaliana*. (a) Schematic diagram for the construction of the OE-*AaBAM3.1* vector. (b) Transgenic *AaBAM3.1* lines seeds in *A. thaliana* were screening with Kanamycin (Kan) (100 mg L-1; Sigma, Japan). (c) Transgenic *AaBAM3.1* lines were screening by DNA level, M, marker DL2000; 1-8, transgenic lines; WT, wild type plants (negative control); CK, H_2_O_2_ (negative control); P, recombinant plasmid (positive control).


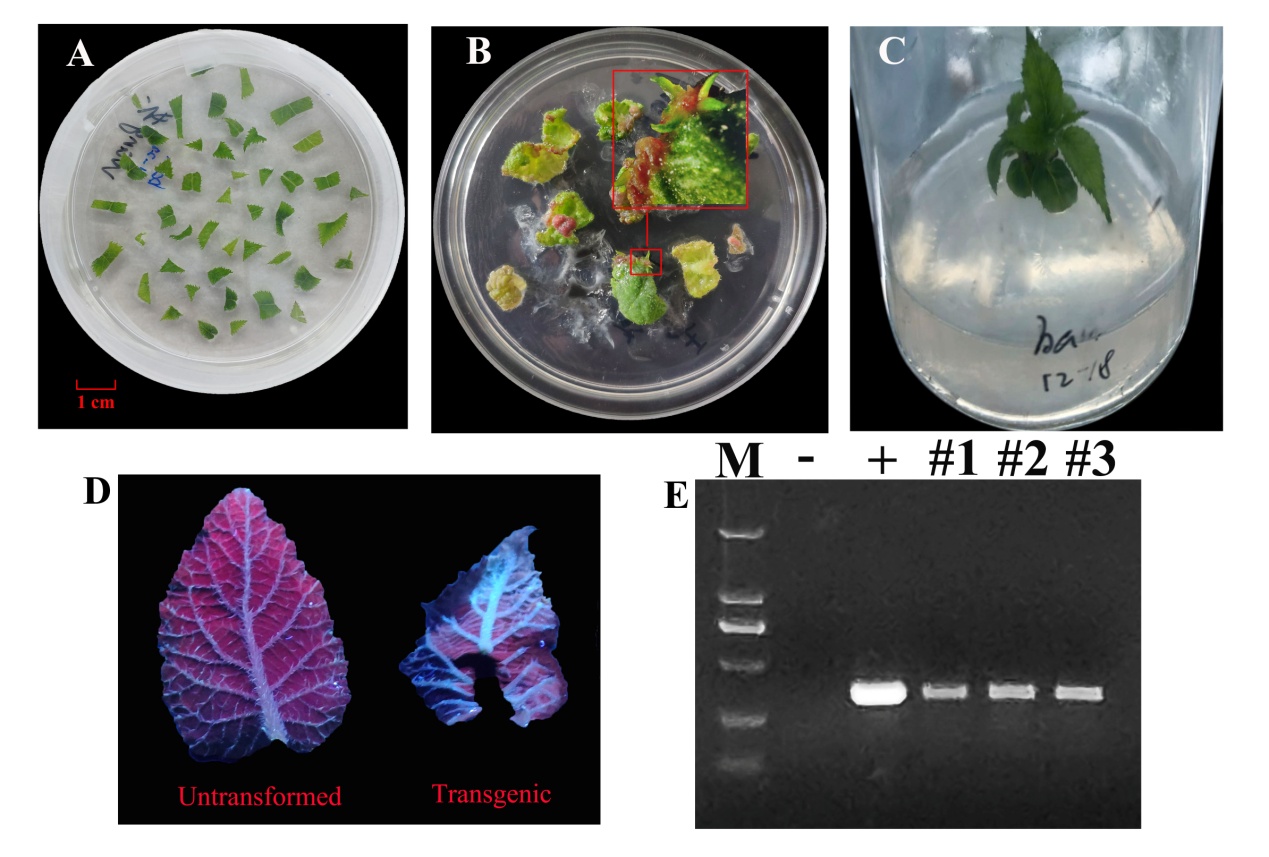


Figure S2. Positive transgenic screening of overexpressed *AaBAM3.1* transgenic lines in *A. chinensis* (cv. ‘Hongyang’). Leaf strips transformation protocol for *A. chinensis* was showned in (A), (B) and (C), respectively. (D) Using reporter gene GFP expressed in the transgenic lines, portable UV machine was used to detect positive plants. (E) Transgenic *AaBAM3.1* lines were screening by DNA level, M, marker DL2000; -, wild type plants (negative control); +, recombinant plasmid (positive control); #1-#3, transgenic lines.


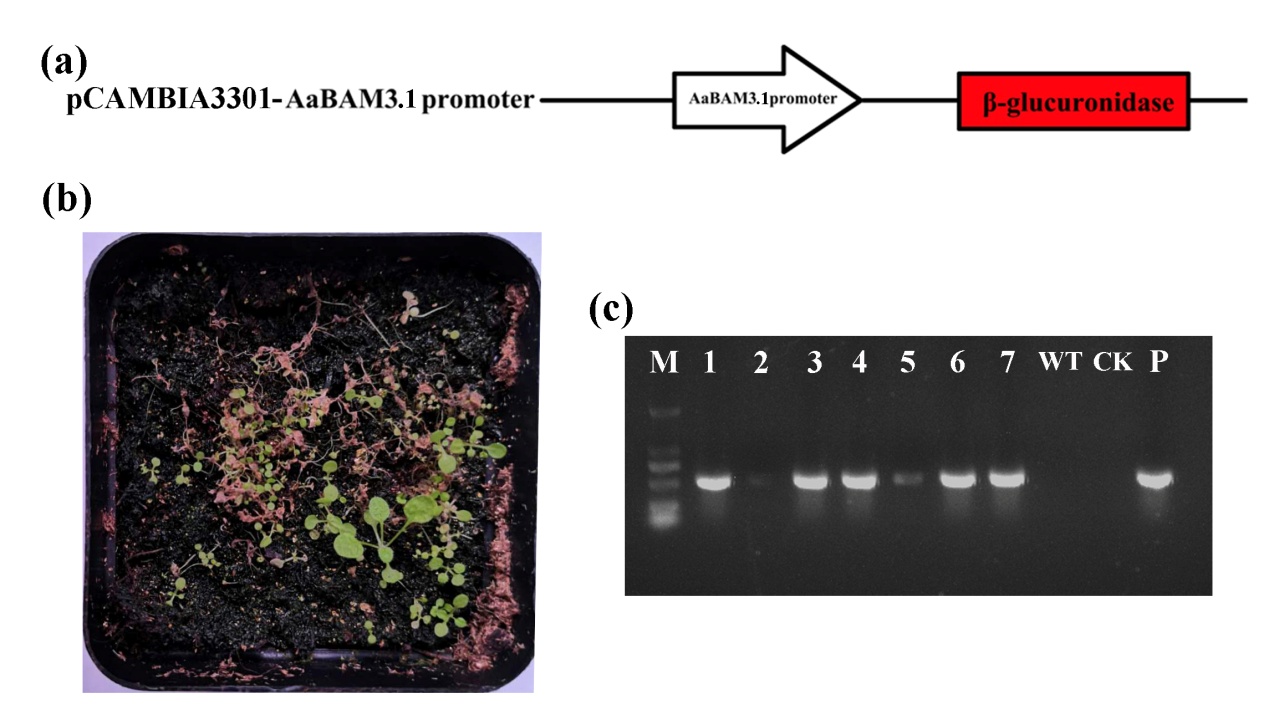


Figure S3. Positive trangenic screening of overexpressed *AaBAM3.1* promoter lines in *A. thaliana*. (a) Schematic diagram for the construction of the *AaBAM3.1* prompter::GUS vector. (b) Transgenic lines seeds in *A. thaliana* were screening with phosphinothricin (Basta) (1 mg L-1; Sigma, Japan). (c) Transgenic *AaBAM3.1* lines were screening by DNA level, M, marker DL2000; 1-7, transgenic lines; WT, wild type plants (negative control); CK, H_2_O_2_ (negative control); P, recombinant plasmid (positive control).


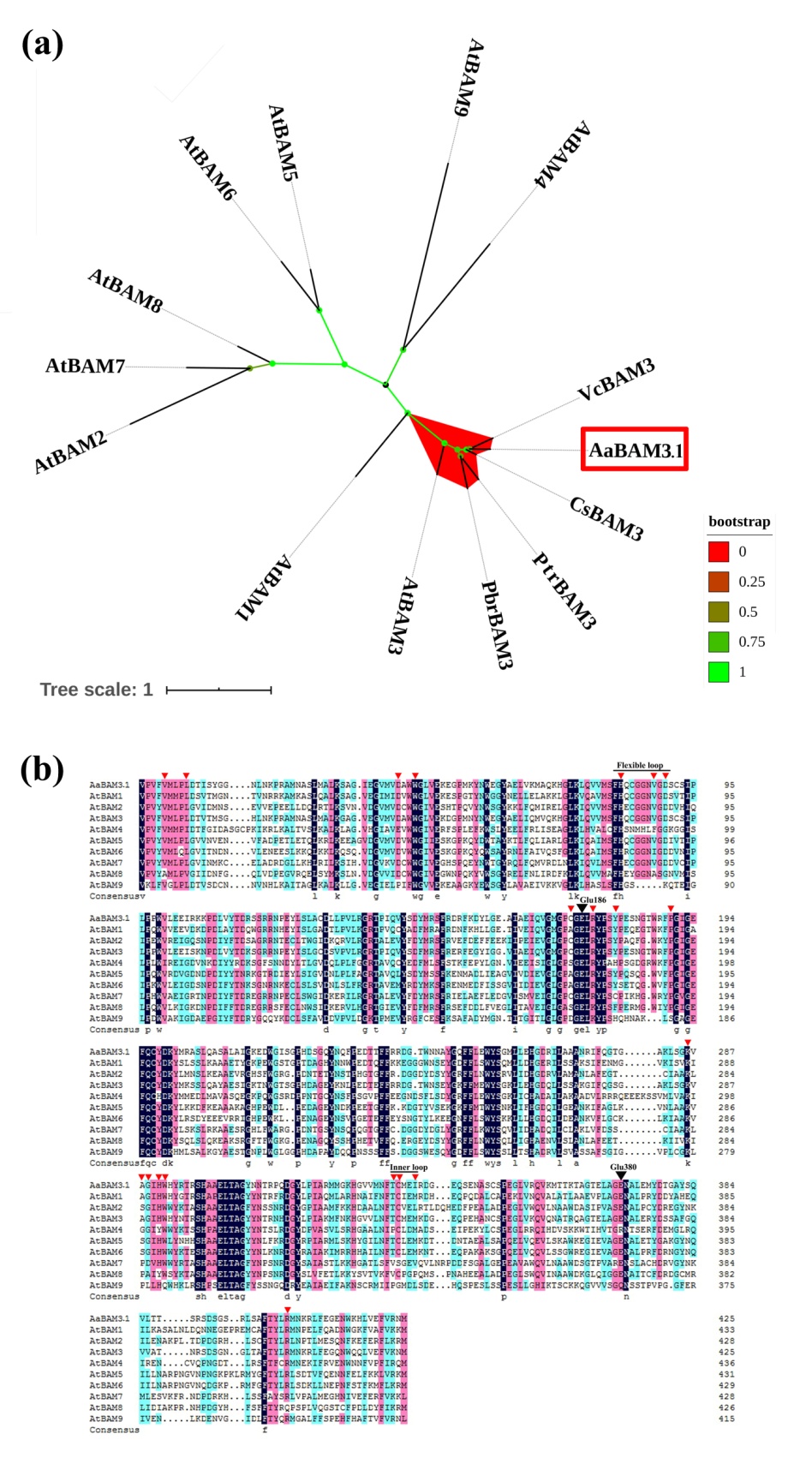


Fig. S4. Characterization of the AaBAM3.1 protein from kiwifruit. (a) The phylogenetic relationships among AaBAM3.1 and homologous proteins. Fourteen BAM protein sequences were obtained from the following 6 species: *A. arguta*, *Camellia sinensis*, *Citrus trifoliata*, *Vaccinium corymbosum*, *Pyrus bretschneideri* and *Arabidopsis*. The GenBank accession numbers of the included BAM proteins were as follows: AaBAM3.1, MT263012; CsBAM3, AHJ09602.1; VcBAM3, AFI56496.1; PtrBAM3, AFQ33613.1; PbrBAM3, XP_009371857.1; AtBAM1, NP_189034.1; AtBAM2, NP_191958.3; AtBAM3, NP_567523.1; AtBAM4, NP_568829.2; AtBAM5, NP_567460.1; AtBAM6, NP_180788.2; AtBAM7, NP_182112.2; AtBAM8, NP_199343.1; AtBAM9, NP_197368.1.(b) Protein sequence alignments of AaBAM3.1 and AtBAM1-9. Identical and relatively conserved residues are indicated by black and red shapes, respectively. The locations of the residues are listed on the right.
