# Supplementary material for: The AaCBF4-AaBAM3.1 module enhances freezing tolerance of kiwifruit (Actinidia arguta)
Source: Hortic Res. 2021 May 1;8:97. doi: 10.1038/s41438-021-00530-1 (PMC8087828; doi:10.1038/s41438-021-00530-1)
Supplement: Supplementary file 2 — supplemental table [file 41438_2021_530_MOESM2_ESM.docx]

Table S1 Primers used in this study

| Assay | Primer sequence | | | Restriction Site |
| --- | --- | --- | --- | --- |
|  |  | Forward primer(5’-3’) | Reverse primer(5’-3’) |  |
| PCR | AaBAM3.1-ORF | ATGGCTTTGGCGCTACATTC | CAGTAGAGCAGCCTCCTTCG |  |
|  | AaCBF1.1-ORF | ATGATGAACCTTGAAGATGATTC | TTAGTATCTCCACAGAGTCATGTCC |  |
|  | AaCBF2.1-ORF | ATGAACTCCGAAGACGAGTCTTCTA | TCACCACAAAAGCAAGTCCATGTGG |  |
|  | AaCBF2.2-ORF | ATGGATGCTTCAAATCCATTCTTCG | TTAGTAACTCCATAATGAAACCTCA |  |
|  | AaCBF4-ORF | ATGGATGCTTCAAATCCATTCTTCG | GTAACTCCATAATGAAACCTCA |  |
| RT-qPCR | AaBAM3.1-Q | ACCTACCAATAGCGCGGATG | AACTAACCCTTCTGGCGAGC |  |
|  | AaCBF4-Q | CAGATTGCACGCATTCCGAG | CGCAAACCCACTTGTCTGTG |  |
| Subcelluar localization | PBI21-GFP- AaBAM3.1 | tgctctagaATGGCTTTGGCGCTACATTC | cgcggatccCAGTAGAGCAGCCTCCTTCG | XbaI ,BamHI |
|  | PBI21-GFP- AaCBF4 | cgcggatccATGGATGCTTCAAATCCATTCTTCG | tcccccgggggaGTAACTCCATAATGAAACCTCA | BamHI, SmaI |
| YIH | PABAi-AaBAM3.1 | cgagctcacCCAAAAACAAAAACTATCAAAAT | ccgctcgagGAAAAAGAAAAATGAACCTCCTCGG | SacI, XhoI |
|  | AD-AaCBF4 | cgcggatccATGGATGCTTCAAATCCATTCTTCG | ccgctcgagTTAGTAACTCCATAATGAAACCTCA | BamHI, XhoI |
| Dual LUC assay | AaBAM3.1 | ggtaccgggccccccctcgagTCGTCGAGAGAGATGAAGATATAAGG | tgtttttggcgtcttccatggATTATGTTTCTTCTTGGTTTTGTGAAG | NcoI  KpnI |
| GUS assay | AaBAM3.1 | caaagggtaatatccggaAACCAAAGCATCTATTTTAATCAGC | ccctcagatctaccatggATCGTTTCTTCTTGGTTTTGTGA | BspEI  NcoI |
|  |  |  |  |  |
